# Supplementary material for: Phylogenomics reveals an almost perfect polytomy among the almost ungulates (Paenungulata)
Source: bioRxiv. 2023 Dec 8:2023.12.07.570590. Preprint. [Version 1] doi: 10.1101/2023.12.07.570590 (PMC10723481; doi:10.1101/2023.12.07.570590)
Supplement: 1 [file NIHPP2023.12.07.570590V1-supplement-1.pdf]

bioRxiv preprint doi: https://doi.org/10.1101/2023.12.07.570590; this version posted December 8, 2023. The copyright holder for this preprint (which was not certified by peer review) is the author/funder, who has granted bioRxiv a license to display the preprint in perpetuity. It is made available under aCC-BY 4.0 International license.

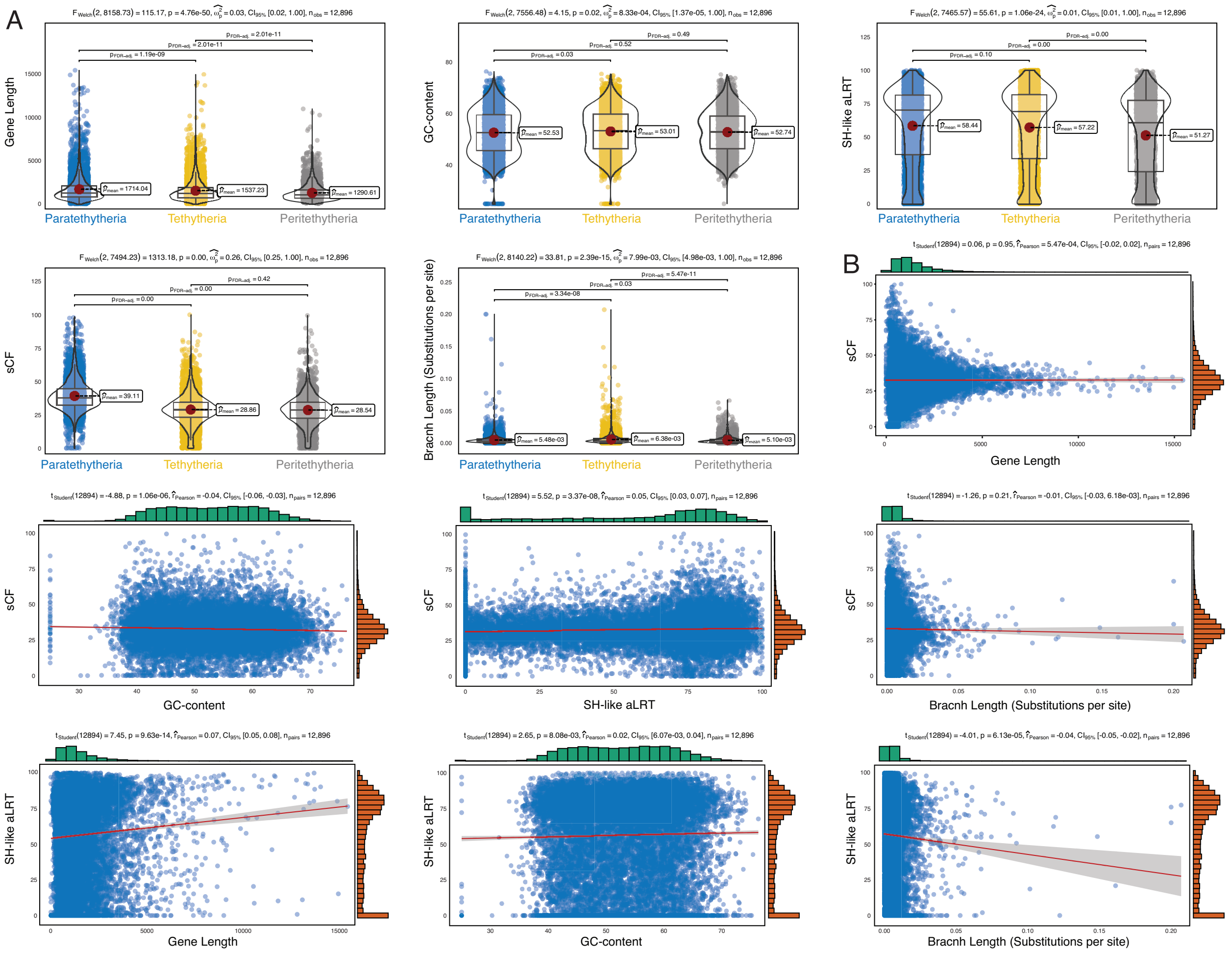

Supplementary Figure 1. Other sources of phylogenetic discordance among *Paenungulates*.

A. Stripchart/violin/boxplots showing differences in mean gene length, GC-content, SH-like aLRT score, sCF, and branch length between genes with gene trees that support the *Paratethytheria*, *Tethytheria*, and *Pertethytheria* splits. Summary statistics and FDR-adjusted *P*-values from pair-wise Games-Howell tests are shown.

B. Scatterplots showing the correlation between variables in panel A; sCF, SH-like aLRT, and branch length were calculated from the ASTRAL species with the *Paratethytheria* split. Summary statistics from Pearson's correlation coefficients are shown above reach split, and side histograms show the distribution of values for each gene.

bioRxiv preprint doi: https://doi.org/10.1101/2023.12.07.570590; this version posted December 8, 2023. The copyright holder for this preprint (which was not certified by peer review) is the author/funder, who has granted bioRxiv a license to display the preprint in perpetuity. It is made available under aCC-BY 4.0 International license.

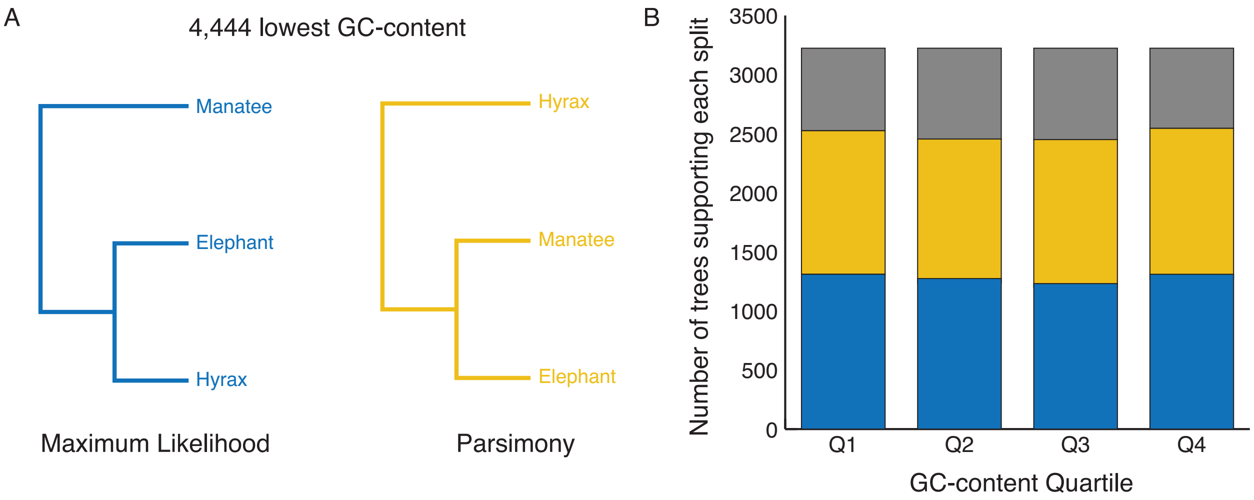

Supplementary Figure 2. GC content variation across genes is an unlikely source of gene tree discordance.

A. Maximum likelihood and parsimony trees inferred from a concatenated supermatrix of the third of genes with the lowest GC content (n=4,444). A GTR substitution model was used for each gene partition in the supermatrix.

B. Number of gene trees supporting each split when genes are binned by GC content quartiles. The *Paratethytheria* split is supported by the greatest number of gene trees in each quartile.

bioRxiv preprint doi: https://doi.org/10.1101/2023.12.07.570590; this version posted December 8, 2023. The copyright holder for this preprint (which was not certified by peer review) is the author/funder, who has granted bioRxiv a license to display the preprint in perpetuity. It is made available under aCC-BY 4.0 International license.

Supplementary Table 1. Molecular phylogenetic studies that have included *Paenungulata*. If the study has more than 10 genes, total alignment length (TAL) is shown.

Study	Data type	Method	Phylogeny
(Weitz, 1953)	Albumin	Antibody cross-reactivity	Paenungulata[4]
(Jong et al., 1977)	Protein (n=1)	Overall similarity	Paenungulata[5]
(Jong et al., 1981)	Protein (n=1)	Parsimony	Peritethytheria
(Jong and Goodman, 1982)	Protein (n=1)	Parsimony	Peritethytheria
(McKenna, 1992)	Protein (n=1)	Parsimony	Peritethytheria
(Rainey et al., 1984)	Albumin	Immunological distances	Unresolved
(JONG et al., 1984)	Protein (n=1)	Parsimony	Peritethytheria
(J. H. Shoshani, 1986)	Albumin	Immunological distances	Paratethytheria
(J. H. Shoshani, 1986)	Whole sera	Immunological distances	Tethytheria
(J. Shoshani, 1986)	Albumin, whole sera	UWPGN	Paratethytheria
(J. Shoshani, 1986)	Morphology	Parsimony	Tethytheria
(Kleinschmidt et al., 1986)	Protein (n=2)	Concatenation, Parsimony	Tethytheria
(Miyamoto and Goodman, 1986)	Protein (n=7)	Concatenation, Parsimony	Paratethytheria
(Miyamoto and Goodman, 1986)	Protein (n=7)	Concatenation, Parsimony	Unresolved
			Tethytheria
(Springer and Kirsch, 1993)	Mitochondrial gene (n=1)	Parsimony	Tethytheria
(Porter et al., 1996)	Nuclear gene (n=1)	Parsimony, Neighbor-joining	Peritethytheria
(Lavergne et al., 1996)	rRNA (n=1)	Parsimony, Neighbor-joining	Tethytheria

[4] Included ox, African elephant, rock hyrax, white rhinoceros, and minke whale but did not include manatee, thus it could not resolve relationships within Paenungulata but noted that African elephant and rock hyrax had several amino acid changes in common compared to the other species.

[5] Included Asian elephant, yellow-spotted rock hyrax (*H. b. prittwitzi*),human, ox, sheep, horse, pig, dog and cat but did not include manatee, thus it could not resolve relationships within Paenungulata but noted that African elephant and rock hyrax anti-elephant and anti-hyrax sera cross-reacted with each other but not the other species.

bioRxiv preprint doi: https://doi.org/10.1101/2023.12.07.570590; this version posted December 8, 2023. The copyright holder for this preprint (which was not certified by peer review) is the author/funder, who has granted bioRxiv a license to display the preprint in perpetuity. It is made available under aCC-BY 4.0 International license.

Study	Data type	Method	Phylogeny
(Stanhope et al., 1996)	Nuclear gene (n=1)	Parsimony, Neighbor-joining	Paratethytheria
(Graur et al., 1997)	Mitochondrial and nuclear genes (n=36, TAL=7.9kb)	Concatenation, Maximum likelihood	Unresolved
(Graur et al., 1997)	Mitochondrial and nuclear genes (n=36, TAL=7.9kb)	Concatenation, Neighbor-joining	Peritethytheria
(Graur et al., 1997)	Mitochondrial and nuclear genes (n=36, TAL=7.9kb)	Concatenation, Parsimony	Unresolved
(Ozawa et al., 1997)	Mitochondrial gene (n=1)	Neighbor-joining (nucleotide and amino acids)	Tethytheria
(Ozawa et al., 1997)	Mitochondrial gene (n=1)	Parsimony (amino acids)	Peritethytheria
(Stanhope et al., 1998)	Nuclear gene (n=1)	Parsimony	Tethytheria
(Stanhope et al., 1998)	Mitochondrial gene (n=2)	Parsimony	Peritethytheria
(Stanhope et al., 1998)	Nuclear gene (n=1)	Parsimony	Paratethytheria
(Stanhope et al., 1998)	Nuclear gene (n=1)	Parsimony	Peritethytheria
(Stanhope et al., 1998)	Nuclear gene (n=1)	Parsimony	Peritethytheria

bioRxiv preprint doi: https://doi.org/10.1101/2023.12.07.570590; this version posted December 8, 2023. The copyright holder for this preprint (which was not certified by peer review) is the author/funder, who has granted bioRxiv a license to display the preprint in perpetuity. It is made available under aCC-BY 4.0 International license.

Study	Data type	Method	Phylogeny
(Noro et al., 1998)	Mitochondrial genes (n=2)	Neighbor-joining, Parsimony	Tethytheria
(Springer et al., 1999)	Mitochondrial and nuclear genes (n=8, TAL=7.3kb)	Concatenation, Maximum likelihood	Paratethytheria
(Springer et al., 1999)	Mitochondrial genes (n=4, TAL=3.3kb)	Concatenation, Maximum likelihood	Tethytheria
(Springer et al., 1999)	Mitochondrial and nuclear genes (n=4, TAL=4kb)	Concatenation, Maximum likelihood	Paratethytheria
(Liu and Miyamoto, 1999)	Nuclear genes (n=3, TAL=3.6kb)	Concatenation, Parsimony	Peritethytheria
(William J. Murphy et al., 2001)	Mitochondrial and nuclear genes (n=22, TAL=16.4kb)	Concatenation, Bayesian	Peritethytheria
(William J. Murphy et al., 2001)	Mitochondrial and nuclear genes (n=22, TAL=16.4kb)	Concatenation, Maximum likelihood	Paratethytheria
(Madsen et al., 2001)	Mitochondrial and nuclear genes (n=6, TAL=2.9kb)	Concatenation, Maximum likelihood	Peritethytheria
(Madsen et al., 2001)	Mitochondrial and nuclear genes (n=6, TAL=5.7kb)	Concatenation, Maximum likelihood	Paratethytheria
(Madsen et al., 2001)	Mitochondrial and nuclear genes (n=7, TAL=8.6kb)	Concatenation, Maximum likelihood	Peritethytheria
(William J Murphy et al., 2001)	Mitochondrial and nuclear genes (n=18, TAL=9.8kb)	Concatenation, Neighbor-joining	Tethytheria
(William J Murphy et al., 2001)	Mitochondrial and nuclear genes (n=18, TAL=9.8kb)	Concatenation, Parsimony	Paratethytheria
(William J Murphy et al., 2001)	Mitochondrial and	Concatenation, Maximum	Tethytheria

bioRxiv preprint doi: https://doi.org/10.1101/2023.12.07.570590; this version posted December 8, 2023. The copyright holder for this preprint (which was not certified by peer review) is the author/funder, who has granted bioRxiv a license to display the preprint in perpetuity. It is made available under aCC-BY 4.0 International license.

Study	Data type	Method	Phylogeny
	nuclear genes (n=18, TAL=9.8kb)	likelihood	
(Delsuc et al., 2002)	Nuclear genes (n=3, TAL=5.1kb)	Concatenation, Maximum likelihood	Peritethytheria
(Waddell and Shelley, 2003)	Mitochondrial and nuclear genes (n=8, TAL=?kb)	Concatenation, Maximum likelihood	Peritethytheria
(Douady and Douzery, 2003)	Nuclear genes (n=3, TAL=1367 AAs)	Bayesian	Tethytheria
(Amrine-Madsen et al., 2003)	Nuclear gene (n=1)	Maximum likelihood, Bayesian	Paratethytheria
(Amrine-Madsen et al., 2003)	Mitochondrial and nuclear genes (n=23, TAL=17.7kb)	Concatenation, Maximum likelihood, Bayesian	Paratethytheria
(Roca et al., 2004)	Mitochondrial and nuclear genes (n=19, TAL=13.9kb)	Concatenation, Maximum likelihood	Peritethytheria
(Nishihara et al., 2005)	Retroposons	Parsimony	Peritethytheria
(Gadagkar et al., 2005)	Mitochondrial and nuclear genes (n=448, TAL=?kb)	Majority rule among gene trees, Neighbor-joining	Paratethytheria
(Nishihara et al., 2006)	Retroposons	Parsimony	Peritethytheria
(Rogaev et al., 2006)	Complete mitochondrial genomes	Bayesian, Parsimony, Neighbor-joining	Peritethytheria
(Murphy et al., 2007)	Mitochondrial and nuclear genes (n=19, TAL=13.9kb)	Concatenation, Maximum likelihood	Peritethytheria
(Kjer and Honeycutt, 2007)	Complete mitochondrial genomes	Concatenation, Bayesian	Tethytheria
(Seiffert, 2007)	Morphology, Nuclear genes (n=23, TAL=17.7kb),	Concatenation, Parsimony	Tethytheria

bioRxiv preprint doi: https://doi.org/10.1101/2023.12.07.570590; this version posted December 8, 2023. The copyright holder for this preprint (which was not certified by peer review) is the author/funder, who has granted bioRxiv a license to display the preprint in perpetuity. It is made available under aCC-BY 4.0 International license.

Study	Data type	Method	Phylogeny
	chromosome painting, and retroposons		
(Asher, 2007)	Morphology, Nuclear genes (n=23, TAL=17.7kb), indels	Concatenation, Parsimony	Tethytheria
(Tabuce et al., 2007)	Morphology	Parsimony	Tethytheria
(Pardini et al., 2007)	Chromosome painting	Parsimony	Unresolved
(Bininda-Emonds et al., 2007)	Supertree	Matrix Representation with Parsimony	Peritethytheria
(Poux et al., 2008)	Nuclear genes (n=4, TAL=4.3kb)	Concatenation, Maximum likelihood	Peritethytheria
(Nishihara et al., 2009)	Retroposon insertions	Parsimony	Peritethytheria
(Poulakakis and Stamatakis, 2010)	Mitochondrial and nuclear genes (n=57, TAL=32.2kb), chromosome rearrangements, and retroposons	Concatenation, Maximum likelihood, Bayesian	Tethytheria
(Kuntner et al., 2011)	Mitochondrial and nuclear genes (n=9, TAL=?kb)	Concatenation, Bayesian	Tethytheria
(Meredith et al., 2011)	Mitochondrial and nuclear genes (n=26, TAL=35.6kb)	Concatenation, Maximum likelihood, Bayesian	Paratethytheria
(O'Leary et al., 2013)	Morphology and nuclear genes (n=27, TAL>35.6kb)	Concatenation, Parsimony	Tethytheria
(Morgan et al., 2014)	Mitochondrial protein coding genes (n=13, TAL= 3906 AA)	Concatenation, Maximum likelihood	Tethytheria

bioRxiv preprint doi: https://doi.org/10.1101/2023.12.07.570590; this version posted December 8, 2023. The copyright holder for this preprint (which was not certified by peer review) is the author/funder, who has granted bioRxiv a license to display the preprint in perpetuity. It is made available under aCC-BY 4.0 International license.

Study	Data type	Method	Phylogeny
(Wu et al., 2014)	Mitochondrial protein coding genes (n=12, TAL=10.7kb)	Concatenation, Maximum likelihood	Tethytheria
(Springer et al., 2015)	Nuclear genes (n=26, TAL=30kb)	Concatenation, Maximum likelihood	Tethytheria
(Phillips, 2016) replication of Meredith et al. (2011)	Mitochondrial and nuclear genes (n=26, TAL=35.6kb)	Concatenation, Bayesian	Paratethytheria
(Liu et al., 2017)	Nuclear genes (n=4388, TAL=13,040kb)	Multispecies coalescence (STAR, NJist)	Tethytheria
(Maswanganye et al., 2017)	Mitochondrial gene (n=1)	Bayesian	Tethytheria
(Springer et al., 2019)	Morphology (4,541 characters)	Parsimony on a molecular backbone	Tethytheria
(Upham et al., 2019)	Nuclear genes (n=31, TAL=39,099bp)	“backbone-and-patch”	Paratethytheria
(Vazquez and Lynch, 2021)	Gene duplications	Maximum likelihood	Tethytheria
(Souza et al., 2021)	Mitogenome	Maximum likelihood	Tethytheria
(Álvarez-Carretero et al., 2022)	Nuclear genes (n=15,268, TAL=33.2Mb)	Bayesian	Tethytheria
(Damas et al., 2022)	Reconstructed ancestral karyotypes	Parsimony	Peritethytheria
(Foley et al., 2023)	411,110 genome-wide nearly neutral sites; 100-kb alignment windows along human Chr1, Chr21, Chr22, and ChrX	Multispecies coalescence (SVDQuartests)	Paratethytheria
(Liu et al., 2023)	Dataset A: Nuclear genes (n=26, TAL=35,063bp) Dataset B: Nuclear	Maximum likelihood, Bayesian, Multispecies coalescence (ASTRAL-III)	Paratethytheria

bioRxiv preprint doi: https://doi.org/10.1101/2023.12.07.570590; this version posted December 8, 2023. The copyright holder for this preprint (which was not certified by peer review) is the author/funder, who has granted bioRxiv a license to display the preprint in perpetuity. It is made available under aCC-BY 4.0 International license.

Study	Data type	Method	Phylogeny
	genes (n=98, TAL=67, 080bp)		
Lynch Unpublished	Gene losses	Dollo parsimony	Paratethytheria
